# An Update on Mitochondrial Ribosome Biology: The Plant Mitoribosome in the Spotlight

**DOI:** 10.3390/cells8121562

**Published:** 2019-12-03

**Authors:** Artur Tomal, Malgorzata Kwasniak-Owczarek, Hanna Janska

**Affiliations:** Department of Cellular Molecular Biology, Faculty of Biotechnology, University of Wroclaw, 50-383 Wroclaw, Poland; artur.tomal2@uwr.edu.pl (A.T.); malgorzata.kwasniak-owczarek@uwr.edu.pl (M.K.-O.)

**Keywords:** mitochondrial ribosome, ribosomal proteins, ribosomal rRNA, PPR proteins, translation, plant mitoribosome

## Abstract

Contrary to the widely held belief that mitochondrial ribosomes (mitoribosomes) are highly similar to bacterial ones, recent experimental evidence reveals that mitoribosomes do differ significantly from their bacterial counterparts. This review is focused on plant mitoribosomes, but we also highlight the most striking similarities and differences between the plant and non-plant mitoribosomes. An analysis of the composition and structure of mitoribosomes in trypanosomes, yeast, mammals and plants uncovers numerous organism-specific features. For the plant mitoribosome, the most striking feature is the enormous size of the small subunit compared to the large one. Apart from the new structural information, possible functional peculiarities of different types of mitoribosomes are also discussed. Studies suggest that the protein composition of mitoribosomes is dynamic, especially during development, giving rise to a heterogeneous populations of ribosomes fulfilling specific functions. Moreover, convincing data shows that mitoribosomes interact with components involved in diverse mitochondrial gene expression steps, forming large expressosome-like structures.

## 1. Introduction

The endosymbiotic theory maintains that mitochondria evolved by the engulfment of an alpha-proteobacterium by a eukaryotic progenitor cell [1]. In the course of evolution, most of the alpha-proteobacterial genes have been lost or migrated to the host nucleus and therefore only a small number of genes are now found in the mitochondrial genome [2]. Mitochondria possess not only their own genome but also distinct gene expression machinery comprised of several assemblies, including mitochondrial ribosomes (mitoribosomes). Mitoribosomes are responsible for the translation of the essential mitochondrial mRNAs, such as those coding for components of oxidative phosphorylation (OXPHOS) complexes. Like all ribosomes, the mitoribosome consists of two subunits: a small (SSU) and a large (LSU) one and has subunit-specific structural landmarks like the body, head, and platform of the SSU and the stalks and central protuberance (CP) of the LSU [3]. The SSU binds messenger RNAs (mRNAs) and mediates the interaction between the mRNA codons and the tRNA anticodons on which the fidelity of translation depends [4]. The LSU contains the peptidyl transferase center (PTC) which catalyzes the formation of peptide bonds linking the amino acids delivered by the tRNAs into a polypeptide chain. Both mitoribosomal subunits consist of mitoribosomal RNAs (mtrRNAs) and mitoribosomal proteins (mtRPs) encoded, respectively, by the mitochondrial and mostly by the nuclear genome.

Given the evolutionary origin of mitochondria, the mitoribosomes were expected to be similar to their bacterial counterparts, and early data seemed to confirm this. However, more thorough studies of their composition and structure indicate that in fact they have undergone extensive remodelling. As a consequence, mitoribosomes are not only quite divergent from the bacterial ribosome but also vary significantly between different species [5,6,7,8,9,10,11,12]. This very dynamic evolutionary history of the mitoribosome indicates its uniqueness, since bacterial, cytosolic, and chloroplast ribosomes are much more uniform in terms of their rRNA and protein composition [2]. This review summarizes the current state of knowledge regarding the structure and composition of mitoribosomes as well as their possible spatial connections with other mitochondrial processes in different organisms, with a particular emphasis on plants.

## 2. The Plant Mitoribosome Is Structurally and Compositionally Distinct from both Prokaryotic and Non-Plant Mitochondrial Ribosomes

### 2.1. The Overall Architecture of the Mitoribosome

**Figure 1:**
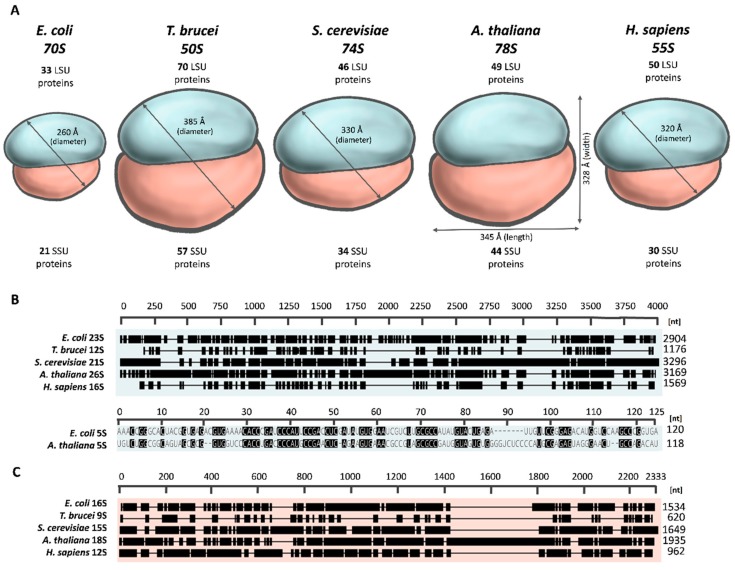
A schematic comparison of size and number of proteins (**A**) as well as rRNAs of the large subunit (LSU) (**B**) and small subunit (SSU) (**C**) of the *E. coli* ribosome and mitoribosomes of *T. brucei*, *S. cerevisiae*, *A. thaliana* and *H. sapiens*. Size and number of proteins are given according to the data from [5,6,7,8,13,17,19]. For the plant mitoribosome, the width and the length were presented since the diameter has not been determined [8]. The alignment of rRNA sequences was made with the MUSCLE (MUltiple Sequence Comparison by Log-Expectation) multiple sequence alignment tool. The 5S rRNA of the LSU is present only in *E. coli* and *A. thaliana***.**

In a cell, ribosomes exist as a mixture of paired and free SSU and LSU subunits whose size is typically referred to in terms of their sedimentation coefficients. The bacterial ribosome has a sedimentation coefficient of 70S [13], while mitoribosomes have been reported to range from 50S in kinetoplastids [14] through 55S in metazoans [15], 74S in fungi [6] to 77–78S in higher plants [16] (Figure 1). Thus, plant mitoribosomes have the highest sedimentation coefficient among all mitoribosomes studied, very close to that of the 80S cytosolic ribosomes. The faster sedimentation rate of plant mitoribosomes results from their increased rRNA and protein content.

The diversity of the mitoribosomes indicated by their different sedimentation coefficients has been convincingly confirmed by cryo-electron microscopy (cryo-EM) studies. So far, the physical dimensions of mitochondrial ribosomes have been determined for *Trypanosomes brucei* [7], *Saccharomyces cerevisiae* [6,17], *Sus scrofa* [15,18], *Homo sapiens* [5,19], and most recently *Arabidopsis thaliana* [8] and *Brassica oleracea* var *botrytis* [16]. With the exclusion of the kinetoplastidic mitoribosomes, the plant mitoribosomes are the largest (345 Å × 328 Å) [8], while the smallest diameter of approximately 320 Å was established for the mammalian mitoribosome [12] (Figure 1). Given their similar sedimentation coefficients, the yeast mitoribosome was expected to be as large as the plant one. However, the lower number of proteins found in the yeast mitoribosome makes them smaller, with a diameter of 330 Å [12]. The other endosymbiotic organelle ribosome—the chlororibosome—is larger compared to the *E. coli* ribosome (by approximately 10 Å along the longest diameter), but smaller than the mitoribosomes [20].

Generally, mitoribosomes differ from bacterial ribosomes by having a higher number of proteins and rRNAs that vary considerably in length across species (Figure 1). The frequency of rRNA indels varies among organisms resulting in more numerous extensions of rRNAs in yeast and plants and a higher abundance of reductions in trypanosomes and mammals. The most spectacular feature of the mitoribosomes of *T. brucei* and *A. thaliana* is the size of their small subunits, which in both species are in fact substantially larger than the corresponding mtLSUs, as revealed by cryo-EM reconstructions [7,8]. This makes them unique not only among mtSSUs but also the SSUs of all known ribosomes. The bulkiness of the *Trypanosoma* mtSSU results from the recruitment of numerous organism-specific proteins [7], whereas in *Arabidopsis,* it is related to both acquisition of plant-specific proteins and expanded rRNAs [8]. A recent cryo-EM reconstruction has revealed several structural features of the *Arabidopsis* mtSSU, namely its large and elongated additional head domain, a distinctive body protuberance and an elongated foot [8]. The distinct architecture of the plant mtSSU foot and head is mainly caused by specific insertions in mtrRNA while the body protuberance is formed predominantly by the mitoribosome-specific proteins. These features are absent in *Arabidopsis* cytoribosomes or *S. scrofa* mitoribosomes, making the plant mtSSU respectively, 1.4-fold and 1.5-fold higher than the SSUs of these two types of ribosomes. A comparative analysis of the *Arabidopsis* and yeast mtSSUs has revealed an absence of the foot and head extensions in yeast mitoribosomes. Similar to the mtSSU, the overall shape of the *Arabidopsis* mtLSU is also strongly remodeled by mitochondria-specific ribosomal proteins, even though a large portion of the core components are similar to those found in bacteria [8].

### 2.2. Variation in Mitoribosomal rRNAs

Both mtSSU and mtLSU contain a single type of rRNA (Figure 1). The only exception is the plant mtLSU with two types of rRNA (26S and 5S rRNA in *A. thaliana*) [21]. The 5S rRNA is an integral component of bacterial, chloroplast and cytosolic LSUs, believed to aid protein synthesis by stabilizing the ribosome structure [22], but it was lost in the kinetoplastidian, yeast, and mammalian mitoribosomes. Recent reports suggest that the loss of 5S rRNA was compensated for functionally in mitochondrial LSUs by rRNA expansion segments and long terminal extensions of several proteins in yeast [12], recruitment of specific proteins in trypanosomes [7], and by transfer RNA (tRNA) in mammals, e.g., tRNA^Val^ in the human or tRNA^Phe^ in the pig [23].

The 26S, 18S and 5S rRNAs of the *Arabidopsis* mitoribosome are, respectively 3169, 1935 and 118 nucleotides (nt) long, making the plant mtSSU and mtLSU rRNAs 20% and 9% larger than their prokaryotic (*E. coli*) counterparts [8] (Figure 1). The 18S rRNA, the structural scaffold for the mtSSU ribosomal proteins contains a 370 nt insertion in helix 39 (h39) of the 3′ major domain of this rRNA located in the head of mtSSU [8]. It is mainly due to this rRNA extension that the plant mtSSU is so big. This expansion is conserved in all angiosperms but may vary in length. For instance, in *Vitis vinifera,* the h39 insertion is longer by 200 nt, while in *Oryza sativa,* it is much shorter than in *A. thaliana.* It has been proposed that this insertion is able to bind plant-specific mitoribosomal proteins, like ribosomal pentatricopeptide repeat proteins (rPPRs) and/or translation factors [8]. Moreover, there are smaller insertions, a 56 nt insertion in h6 of the 5′ domain and a 47 nt insertion in h44 of the 3′ minor domain of 18S rRNA in the foot of mtSSU in *A. thaliana,* which together with the main rRNA insertion in h39 make the plant mtSSU rRNA the longest among all known mitochondrial SSU rRNAs. Interestingly, in yeast, the extensions in the mtSSU rRNA are much shorter [6], but their mtLSU rRNA is the longest of all known mtLSU rRNAs owing to as many as 11 expansions totaling 392 nucleotides [17].

In contrast to the insertional modifications in plants and yeast, in mammals and kinetoplastids, the rRNAs of both mitoribosomal subunits underwent numerous deletions. As a consequence, they are approximately two and even three times shorter, respectively, than their bacterial counterparts [7,15] (Figure 1). In the mammalian mitoribosomes, the largest reductions are in two domains of the mtLSU rRNA and the mtrRNA is limited to the innermost core of the ribosome encompassing the functionally important sites, particularly the PTC active site and the decoding site [15,18]. Accordingly, the mitoribosomes of kinetoplastids have the shortest rRNAs among all organisms. Also the rRNAs of plant [8,16] and yeast [6] mitoribosomes show deletions, but less extensive than those in mammals or trypanosomes.

Taken together, since the same tendency for mtrRNA length changes is observed in highly diverged eukaryotes (yeast and plants on one hand and trypanosomes and mammals on the other), it appears that there was no single dominant evolutionary trend regarding the size of mtrRNA. It should be emphasized that the mtSSU rRNA of kinetoplastids, yeast, plants and mammals lacks the anti-Shine–Dalgarno sequence, the sequence present at the 3′-end of prokaryotic SSU rRNA which facilitates the binding between the ribosome and mRNA, suggesting that protein synthesis in mitochondria does not rely on bacterial-like translation initiation. In contrast, the anti-Shine–Dalgarno sequence is conserved in the SSU rRNA of the chlororibosome [24]. Moreover, chloroplast rRNAs are similar in length to their *E. coli* counterparts [25].

### 2.3. Variation in Mitoribosomal Protein Composition

In all the species examined, the number of mitoribosomal proteins is much greater than the number of proteins identified in bacterial ribosomes (54 for *E. coli*) (Figure 1). A combination of various proteomic approaches has produced lists of 127, 94, 80 and 80 mitoribosomal proteins in *T. brucei, A. thaliana, S. cerevisiae* and *H. sapiens,* respectively (Figure 1) [5,6,7,8,11]. In this context, it seems interesting that chlororibosomes have not acquired as many proteins (only five) as the mitoribosomes carry only a total of 57 ribosomal proteins [25]. In the protein-rich mitoribosome, the protein–protein network shows much higher interconnectivity than that found in the bacterial ribosome [26]. For instance, the yeast and mammalian mtLSU proteins typically have four or five neighbors, while the average bacterial protein is adjacent to only 1.5 neighbors [5,19].

Numerous mitoribosomal proteins are derived from the ancestral alpha-proteobacterial endosymbiont, while others have been recruited during evolution from proto-eukaryotic host cells [2,9]. The mitoribosome of the last eukaryotic common ancestor (LECA), after the establishment of the proto-mitochondrion, had an α-proteobacterial core consisting of 20 SSU and 33 LSU proteins supplemented, respectively, by 10 (S23, S25, S29, S33, S34, S35, S36, Rsm22, Ppe1, Mrp10) and nine (L38, L41, L43, L45, L46, L49, L52, L53, L54) additional, eukaryote-specific proteins [2]. Subsequently, it underwent dynamic evolutionary changes in different eukaryotic lineages, involving a loss of different sets of ribosomal protein-coding genes, their transfer to the host nuclear genome, as well as the acquisition of many novel components. The endosymbiotic transfer of ribosomal genes from mitochondria to the nucleus has reached its limit in animals since all these genes are now nuclear [27]. Similarly, all yeast mitoribosomal proteins, with the single exception of a protein of the small subunit (uS3m, also known as Var1), are nuclear-encoded [28]. However, in higher plants the ribosomal gene transfer to the nucleus continues [29]. The most frequent mitochondrial gene loss is observed for two genes, *rps10* and *rpl2,* which have both undergone remarkably numerous independent gene transfer events [29,30]. The number of the ribosomal proteins still encoded in mitochondria varies among plant species, the bryophyte *Marchantia polymorpha* having the most (16) and *Arabidopsis* having only seven [30].

Most of the 54 proteins of the present-day bacterial ribosome have counterparts in the *Arabidopsis* mitoribosome [8], but the plant proteins are significantly larger, containing additional domains likely to be performing extra functions [30]. Taken together, 94 *Arabidopsis* proteins (49 in mtLSU, 44 in mtSSU and one of ambiguous provenance) have been identified (Table 1) [8]. It should be emphasized that these numbers represent all sequence isoforms found. If a single protein species is considered for each function (some mitoribosomal proteins are encoded by more than one paralogue) the *Arabidopsis* mitochondrial ribosome contains 83 unique proteins. Among them, 51 show clear-cut homology to bacterial ribosomal proteins, 13 to those found in yeast and/or animal mitoribosomes, and 19 are unique to *Arabidopsis* (Table 1). The universally conserved and mitochondria-specific ribosomal proteins form the common protein core of almost all mitoribosomes known, thus confirming their acquisition early during evolution of the eukaryotic cell [16].

Among the organism-specific supernumerary mitoribosomal proteins are ribosomal PPRs (rPPRs). Given the fact that the PPRs constitute one of the largest protein families in plants [31], it is not surprising that their share in the mitoribosome is the highest in plants (10 rPPRs in *A. thaliana*) [8]. rPPRs are also found in mitoribosomes in kinetoplastids (six rPPRs) and mammals (two rPPRs) [5,7]. In contrast, studies of the yeast mitoribosome have not identified any PPR proteins. It is noteworthy that most of the plant rPPRs appear to interact with the large rRNA expansion segments, suggesting their participation in rRNA stabilization [16]. As part of the plant mitoribosome, rPPR proteins may help in the recruitment and docking of mRNA to the mtSSU. For instance, it has been postulated that the plant rPPR protein mS83 recognizes the A/purine-rich sequence AxAAA located in the 5′ untranslated region (UTR)about 19 nt upstream of the AUG codon, thereby facilitating the correct positioning of the mRNA during translation initiation [16]. It is important to note that this A-rich *cis*-element is absent in some plant mitochondrial mRNAs, therefore other as yet unknown translation initiation mechanisms must operate in plant mitochondria. In turn, *Arabidopsis* rPPR mS76 (also known as rPPR1 or PPR336) has been shown to be a generic translation factor required for optimal translation levels of most, if not all, mitochondrial mRNAs. A general impact on mitochondrial translation has also been documented for two mammalian rPPRs known as mS39 and mS27 [32,33]. Knock-down of both mS39 and mS27 results in an overall decrease in translation of mitochondrially-encoded proteins and consequently strongly decreases the abundance of respiratory complexes and mitochondrial respiration [32,33]. In contrast, studies on the rPPR function in kinetoplastids have revealed that two rPPRs specific to *T. brucei*, mS51 and mS55 (also known as kinetoplast ribosomal PPR proteins - KRIPP1 and KRIPP8, respectively), act as selective translational activators for two distinct but partially overlapping subsets of mitochondrial mRNAs [34]. Furthermore, studies in *Arabidopsis* and *Trypanosoma* indicate the indispensability of rPPRs for proper growth and viability. A deficiency of any of the three rPPR proteins, mL101-rPPR4, mL102-rPPR5 or mL103-rPPR7, in *Arabidopsis* delays seedling growth, whereas a knock-down of mS77 or mS79 (also known as rPPR2 and rPPR3b) is lethal or leads to an inability to produce seeds, respectively [8]. In *T. brucei,* the organism-specific mS51 and mS55 are necessary for viability of its pro-cyclic form [34].

### 2.4. Association of Mitoribosomes with the Inner Mitochondrial Membrane

In plant, yeast and mammalian cells, the mitoribosomes have been found attached to the inner mitochondrial membrane (IMM), implying that mitochondrial translation is carried out by membrane-bound ribosomes [35]. In yeast and mammals, all mitochondrial translation products, with the exception of the yeast mitoribosomal protein Var1, are hydrophobic. Nevertheless, it has been proposed that the translation of soluble Var1 also occurs at or in close proximity to the inner membrane [36]. In contrast to mammals and yeast, several hydrophilic proteins are synthesized in plant mitochondria (e.g., seven soluble ribosomal proteins are encoded mitochondrially in *Arabidopsis*). Thus, it is not clear whether all mitochondrially-encoded proteins are indeed synthesized on membrane-bound mitoribosomes in plants or whether a distinct pool of plant mitoribosomes, less tightly associated with the IMM, is responsible for the synthesis of at least some of the soluble proteins. In this context, it is interesting that in yeast, mammals and trypanosomal mitoribosomes that the peptide exit channel is highly remodeled by the species-specific proteins while in plants it is rather bacterial-like, with only minimal rearrangement [8]. Recent cryo-EM studies of plant mitoribosomes suggest that at least in some cases, a non-ribosomal protein links the mitoribosome to the mitochondrial oxidase assembly protein 1 (Oxa1) insertase [16]. In agreement with this, Oxa1 has been found to co-purify with immuno-precipitated *Arabidopsis* mitoribosomes [8]. Cryo-EM reconstruction of the yeast mitoribosome has revealed two distinct membrane contact sites formed, respectively, by the mitochondrial inner membrane protein Mba1 and the expansion segment (96-ES1) in the mtLSU rRNA [37]. The mtrRNA-mediated contact site of the mitoribosome with IMM is absent in mammals while the human homologue of Mba1 (mL45) integrated in the large ribosomal subunit creates a single major contact site with the inner membrane [38].

## 3. Heterogeneity of the Mitoribosome Composition

Ribosomes have long been viewed as homogeneous assemblies, each formed according to identical specifications, ensuring translation of each mRNA with the same efficiency. However, studies across species have not supported the concept of uniform ribosomes, providing ample evidence for their heterogeneity due to a diversity in the mtrRNA sequence and its modifications, variable ribosomal protein content and post-translational modifications, as well as the presence of diverse ribosome-associated factors [39,40,41]. In contrast to the fairly well-established heterogeneity of bacterial and eukaryotic cytoribosomes [40,42], reports supporting such heterogeneity for mitochondrial ribosomes are sparse and limited to alterations in their protein composition [43].

One of the sources of mitoribosome heterogeneity, particularly in plants, is the presence of mtRP gene paralogues forming small multigenic families derived from a single ancestral gene. In *Arabidopsis*, 16 mtRPs are encoded by such small multigenic families [43]. Each mtRP in the mitoribosome is represented by a single polypeptide, thus the presence of paralogs encoding various isoforms leads to several distinct pools of mitoribosomes, each pool with a defined isoform. The presence of mtRP paralogs in plants was originally shown for *Solanum tuberosum*, where four paralogs of the mL12 protein differentially associated with mitochondrial ribosomes were identified [35]. Several studies suggest that mitoribosomes containing different mtRP variants may react differently in response to diverse physiological situations during development. For instance, one of eight mL18 paralogs in *Arabidopsis*, encoded by the *HEART STOPPER* (*HES*) gene, is preferentially expressed in tissues with active cell division and differentiation (developing embryo and root tip) [44]. The tissue-type restricted expression of the *HES* gene, the divergence of the mL18 family, and the failure of other mL18 family members to complement the *hes* phenotype suggest the existence of heterogeneous mitoribosomes with specific functions in development. In this context, one should also mention the tissue-specific expression of one of the components of the mtLSU, mL9(DEK44 – defective kernel 44) in *Zea mays* [45]. DEK44 accumulates only in maize kernels, suggesting that mitoribosomes containing this protein are present exclusively in kernels, but not in other tissues.

Formation of unique heterogeneous populations of mitoribosomes has been reported for plant mutants with a decreased amount or absence of a single mtRP. Thus, RNAi-silencing of the mS10 protein in *Arabidopsis* generated a heterogeneous population of mitoribosomes where mitoribosomes with a partially assembled SSU coexisted with wild-type ones and an excess of free LSUs [46]. Another example comes from mammals, where a knock-down of the mL10 protein affected mitochondrial ribosome assembly in a complex manner, as indicated by a significant shift in the sucrose gradient sedimentation profile for several components of the small and large mitoribosomal subunits [47]. Additionally, it has been revealed that the mammalian mL12 protein exists as two (short and long) forms generated by two-step proteolysis, and both forms were found in mitoribosomes [47,48].

In papers from our laboratory [46,49,50] we have shown that the alteration in mitoribosomal protein composition caused by gene silencing can affect the functioning of the mitoribosome. The altered mitoribosomes are able to perform their basic activities (produce proteins), but they differ functionally from the wild-type ones and favor a specific translational program. Specifically, *Arabidopsis* mitoribosomes altered due to the silencing of the nuclear *RPS10* gene encoding mitochondrial ribosomal protein S10 differentially translate two subsets of mRNAs, those encoding OXPHOS subunits and ribosomal proteins with, respectively, slightly lowered and clearly enhanced efficiency compared to the wild-type. A mitoribosome-mediated translation regulation has also been reported for yeast mitoribosome lacking the SSU protein mS38/Cox24 [51]. It was reported that mS38 is preferentially required for translation initiation of *COX1*, *COX2* and *COX3* mRNAs. The mechanism involves functional interactions of the mS38 protein with the 5′ UTRs of the *COX* mRNAs. The results obtained for the *rps10* and Δ*mS38* mutants support the ribosomal filter hypothesis, postulating that ribosomes are not simply translation machines but also function as regulatory elements that differentially affect or filter the translation of particular mRNAs [52]. It can be safely presumed that the other heterogeneous populations of mitoribosomes mentioned above, both induced by genetic manipulation and formed naturally, may also selectively affect mitochondrial translation. However, so far the evidence for a compositional heterogeneity of mitoribosomes is in most cases much stronger than the evidence for their functional specialization.

## 4. Mitoribosome Features Revealed by Ribosomal Profiling

The development of mitoribosome profiling, an experimental approach using deep sequencing of mitochondrial ribosome-protected fragments (RPFs), has allowed revealing aspects of the mitoribosome interactions with the mtmRNA. In yeast, mitochondrial RPFs were around 38 nt long [53], while a bimodal length distribution of RPFs was found in the human [54], maize [55] and *Arabidopsis* [50]. Data for human mitoribosomes showed a major peak at 27 nt and a minor peak at 33 nt while the maize RPFs showed peaks at 28–29 nt and 36 nt. Recent ribosome profiling for *Arabidopsis* indicates that the majority of mitochondrial ribosome footprints measure 28 nt while reads of 31–32 nt are much less frequent [50]. Moreover, it was shown that the *Arabidopsis* mitoribosomes altered due to the S10 protein deficiency protect shorter mRNA fragments, suggesting that the size of the RPFs reflects distinct conformations of the mitoribosomes [50].

A mitoribosomal profiling analysis of the *Arabidopsis* translatome has revealed strikingly different mitoribosome association levels with different mtRNAs. mRNAs encoding subunits of OXPHOS complexes showed much higher ribosome loading levels compared to mitochondrial transcripts of ribosomal proteins or those encoding *c*-type cytochrome maturation factors, implying that the OXPHOS proteins are translated preferentially [50,56]. Low levels of mitoribosome footprints were detected for maturase (MatR) and the TatC protein also known as MttB although these proteins are not identified by mass spectrometry [57]. Interestingly, the altered mitoribosomes in *rps10*, markedly more efficiently synthesized the MatR and TatC proteins [50].

An interaction between partially edited mRNAs and mitoribosomes has also been indicated by the results of mitoribosomal profiling in *Arabidopsis* [56], supporting earlier studies which showed that complete editing of mitochondrial mRNAs is not a pre-requisite for their translation. However, the mitoribosome-associated mRNAs are more fully edited than the total pool of RNAs. All this suggests that a non-negligible proportion of proteins produced in plant mitochondria could derive from partially edited transcripts, but the advantage coming from this feature—if any—is not clear [58]. Notably, our study of the *rps10* mutant of *Arabidopsis* has uncovered an unexpected link between the deficiency of the S10 mitoribosomal protein and mitochondrial splicing. The deficit of S10 shifted the balance between spliced and unspliced transcripts toward the latter. Whether this effect is due to dysfunctional mitoribosomes or is caused by a compromised hypothetical non-ribosomal function of S10 remains to be established.

## 5. Mitoribosomes in Expressosome-Like Assemblies

While in the eukaryotic cell, the nuclear envelope separates cytoplasmic translation from transcription. In mitochondria, these processes occur in close proximity, as they do in bacteria. Studies in mammalian mitochondria have shown that the newly synthesized RNA is concentrated in so called mitochondrial RNA granules [59,60]. These assemblies are not enclosed by lipid bilayers but rather form “droplet organelles” owing to the phase separation phenomenon and are believed to connect transcription with downstream processes such as RNA processing and ribosome biogenesis because numerous proteins involved in these events have also been identified as their components [61]. Among the proteins found in the RNA granules are components of the mitochondrial translation machinery, mtSSU and mtLSU proteins, aminoacyl-tRNA synthetases, and factors involved in ribosome assembly and disassembly [62]. Recently, it was shown that most RNA granules are in close association with nucleoids and occasionally with RNA degradosomes [63]. Thus, it seems that at least in mammalian mitochondria, the nucleoids, RNA granules and protein synthesis machinery are tightly associated with each other.

Similarly, large assemblies containing factors involved in post-transcriptional mRNA maturation, translation and RNA decay have been reported in yeast and termed mitochondrial organization of gene expression (MIOREX) complexes [64]. Interestingly, these structures contain complete mitoribosomes. It has also been reported that proteins engaged in the biogenesis of mitochondrial membrane proteins can be found in MIOREX [65]. Furthermore, a mass-spectrometric analysis has revealed an association between mitoribosomes and the membrane-bound AAA proteases in yeast [66]. Most of the MIOREX complexes are evenly distributed throughout the mitochondrial network, whereas a subset is present as nucleoid–MIOREX complexes that apparently carry out all the steps of mitochondrial gene expression [67].

Compared to mammalian and yeast mitochondria, our knowledge about the mitoribosomal interactome in plants is limited, albeit a recent publication [11] fills this gap partially. Using a new comprehensive profiling strategy in combination with chemical cross-linking, more than 80 proteins have been identified that are part of or associate with the *Arabidopsis* mitoribosome. These proteins also include factors involved in processes preceding (e.g., RNA editing) and following (e.g., polypeptide maturation) translation itself. Given the absence of these proteins on the list of mitochondrial ribosome core proteins published by [8], they were suggested to form only loose assemblies with mitoribosomes. Despite this weak association, upon cross-linking they co-migrate with the mtLSU in large pore Blue-Native polyacrylamide gel electrophoresis (lpBN-PAGE). The identification among them of four PPR-proteins (DYW2, MEF11, MEF29, and AT5G40405) containing a DYW domain, recently confirmed as the cytidine deaminase editing domain [68], indicates a physical connection between the RNA editosome and the mitoribosome. In turn, the detection of two subunits of m-AAA protease (FtsH3 and FtsH10) and two members of the S24/S26 peptidase family suggests that plant mitoribosomes interact with complexes involved in protein processing and degradation.

## 6. Conclusions

While the structure of mitoribosomes in non-plant species has been known for several years, the architecture and protein composition of a plant mitoribosome were determined only recently. It turned out to be one of the largest and certainly the most complex mitoribosome described to date, and the most unexpected finding was that its “small” subunit is in fact larger than its “large” subunit. The unique evolutionary path leading to the present-day plant mitoribosome included large expansions of rRNAs and incorporation of supernumerary proteins. These additional rRNA segments and proteins have reshaped the overall architecture of the plant mitochondrial ribosome. The plant-specific proteins include PPRs which interact with the plant-specific rRNA expansion segments; their functions in translation are only beginning to emerge. Similarly, our understanding of the molecular foundations of the functional diversity of the heterogenous population of mitoribosomes is at best elementary. Future efforts should focus on factors modulating the mitoribosome composition in vivo. Likewise, there is still much to learn about the connections—physical as well as functional—between the translation machinery and the components of the other stages of plant mitochondrial gene expression.

## Figures and Tables

**Table 1:** Comparison of *Arabidopsis thaliana* mitoribosome core proteins with their homologues in bacterial ribosomes and mitoribosomes of kinetoplastids, yeast and mammals. Mitoribosomal proteins (mtRPs) of the small and large subunits and mtRPs whose assignment to either subunit is unclear. The *Arabidopsis* mtRPs are colored to indicate their conservation with the bacterial ribosome (light orange and blue) and non-plant mitoribosomes (medium orange and blue). Proteins found exclusively in *Arabidopsis* are colored dark orange and blue. ^1^ Protein isoforms encoded by a family of paralogous genes. ^2^ The THXm protein has been found only in Thermus spp. bacteria. ^3^ ribosomal pentatricopeptide repeat protein.

Higher Plants (*A. thaliana*) [8]	*Arabidopsis* Gene ID	Bacteria (*E. coli*) [13]	Kinetoplastids (*T. brucei*) [7]	Yeast (*S. cerevisiae*) [6]	Mammals (*H. sapiens*) [5]
Small Subunit					
uS2m	AT3G03600	✓		✓	✓
uS3m	ATMG00090	✓	✓	✓	✓
uS4m	ATMG00290	✓		✓	
uS5m	AT1G64880	✓	✓	✓	✓
bS6m	AT3G18760	✓	✓	✓	✓
uS7m	ATMG01270	✓		✓	✓
uS8m ^1^	AT4G29430 AT2G19720	✓	✓	✓	
uS9m	AT3G49080	✓	✓	✓	✓
uS10m	AT3G22300	✓	✓	✓	✓
uS11m	AT1G31817	✓	✓	✓	✓
uS12m	ATMG00980	✓	✓	✓	✓
uS13m	AT1G77750	✓		✓	
uS14m	AT2G34520	✓	✓	✓	✓
uS15m ^1^	AT1G15810 AT1G80620	✓	✓	✓	✓
bS16m	AT5G56940	✓	✓	✓	✓
uS17m	AT1G49400	✓	✓	✓	✓
bS18m	AT1G07210	✓	✓	✓	✓
uS19m	AT5G47320	✓	✓	✓	
bS21m	AT3G26360	✓	✓	✓	✓
bTHXm ^2^	AT2G21290	✓			
mS22	AT1G64600		✓		✓
mS23	AT1G26750		✓	✓	✓
mS29	AT1G16870		✓	✓	✓
mS33	AT5G44710		✓	✓	✓
mS34	AT5G52370		✓	✓	✓
mS35 ^1^	AT3G18240 AT4G21460		✓	✓	✓
mS47	AT4G31810		✓	✓	
mS75	AT5G62270				
mS76 (rPPR1 ^3^)	AT1G61870				
mS77 (rPPR2 ^3^)	AT1G19520				
mS78 (rPPR3a ^3^)	AT1G55890				
mS79 (rPPR3b ^3^)	AT3G13160				
mS80 (rPPR6 ^3^)	AT3G02650				
mS81 (rPPR8 ^3^)	AT5G15980				
mS82	AT4G22000				
mS83	AT4G15640 AT3G21465				
mS84	AT1G53645				
mS85	AT1G18630				
mS86	AT1G47278				
mS87	AT5G26800				
Large Subunit					
uL1m	AT2G42710	✓		✓	✓
uL2m	AT2G44065	✓		✓	✓
uL3m	AT3G17465	✓	✓	✓	✓
uL4m	AT2G20060	✓	✓	✓	✓
uL5m	ATMG00210	✓		✓	
uL6m	AT2G18400	✓		✓	
bL9m	AT5G53070	✓	✓	✓	✓
uL10m	AT3G12370	✓	✓	✓	✓
uL11m	AT4G35490	✓	✓	✓	✓
bL12m ^1^	AT3G06040 AT1G70190 AT4G37660	✓	✓	✓	✓
uL13m	AT3G01790	✓	✓	✓	✓
uL14m	AT5G46160	✓		✓	✓
uL15m	AT5G64670	✓	✓	✓	✓
uL16m	ATMG00080	✓	✓	✓	✓
bL17m ^1^	AT5G09770 AT5G64650	✓	✓	✓	✓
uL18m	AT5G27820	✓			✓
bL19m	AT1G24240	✓	✓	✓	✓
bL20m	AT1G16740	✓	✓		✓
bL21m	AT4G30930	✓	✓	✓	✓
uL22m ^1^	AT1G52370 AT4G28360	✓	✓	✓	✓
uL23m	AT4G39880	✓	✓	✓	✓
uL24m	AT5G23535	✓	✓	✓	✓
bL25m ^1^	AT4G23620 AT5G66860	✓			
bL27m	AT2G16930	✓	✓	✓	✓
bL28m	AT4G31460	✓	✓	✓	✓
uL29m	AT1G07830	✓	✓	✓	✓
uL30m	AT5G55140	✓	✓	✓	✓
bL31m ^1^	AT5G55125 AT1G27435	✓	✓	✓	
bL33m	AT5G18790	✓	✓	✓	✓
bL36m	AT5G20180	✓	✓	✓	✓
mL40	AT4G05400		✓	✓	✓
mL41 ^1^	AT5G40080 AT5G39800		✓	✓	✓
mL43	AT3G59650		✓	✓	✓
mL46	AT1G14620		✓	✓	✓
mL53	AT5G39600		✓	✓	✓
mL54	AT3G01740			✓	✓
mL101 (rPPR4 ^3^)	AT1G60770				
mL102 (rPPR5 ^3^)	AT2G37230				
mL103 (rPPR7 ^3^)	AT4G36680				
mL104 (rPPR9 ^3^)	AT5G60960				
mL105	AT3G51010				
mL106	AT1G73940				
Assignment to either subunit unclear					
mrpX	AT5G49210				
